# Outbreak of imported diphtheria with *Corynebacterium diphtheriae* among migrants arriving in Germany, 2022

**DOI:** 10.2807/1560-7917.ES.2022.27.46.2200849

**Published:** 2022-11-17

**Authors:** Franziska Badenschier, Anja Berger, Alexandra Dangel, Annika Sprenger, Bernhard Hobmaier, Claudia Sievers, Henrieke Prins, Achim Dörre, Christiane Wagner-Wiening, Wiebe Külper-Schiek, Ole Wichmann, Andreas Sing

**Affiliations:** 1 Department of Infectious Disease Epidemiology, Postgraduate Training for Applied Epidemiology, Robert Koch Institute (RKI), Berlin, Germany; 2 ECDC Fellowship Programme, EPIET Associated Programme, European Centre for Disease Prevention and Control (ECDC), Stockholm, Sweden; 3 National Consiliary Laboratory for Diphtheria, Bavarian Health and Food Safety Authority, Oberschleißheim, Germany; 4 Unit of Bacteriology, Public Health Microbiology, Bavarian Health and Food Safety Authority (LGL), Oberschleißheim, Germany; 5 NGS Core Unit, Public Health Microbiology, Bavarian Health and Food Safety Authority (LGL), Oberschleißheim, Germany; 6 Department of Infectious Disease Epidemiology, Robert Koch Institute, Berlin, Germany; 7 ECDC Fellowship Programme, Field Epidemiology path (EPIET), European Centre for Disease Prevention and Control (ECDC), Stockholm, Sweden; 8 State Health Office Baden-Wuerttemberg, Stuttgart, Germany

**Keywords:** cgMLST, *Corynebacterium diphtheriae*, diphtheria, infectious disease outbreaks, migration and health, Next Generation Sequencing, outbreak investigation, population surveillance, skin lesions, vaccine-preventable disease

## Abstract

From July 2022, cases of imported diphtheria with toxigenic *Corynebacterium diphtheriae* remarkably increased among migrants arriving in Germany. Up to 30 September 2022, 44 cases have been reported to the national public health institute, all laboratory-confirmed, male, and mainly coming from Syria (n = 21) and Afghanistan (n = 17). Phylogeny and available journey information indicate that most cases (n = 19) were infected along the Balkan route. Active case finding, increased laboratory preparedness and epicentre localisation in countries along this route are important.

Since the end of July 2022, a remarkable increase of cases of imported, mainly cutaneous diphtheria caused by toxigenic *Corynebacterium diphtheriae* has been observed among migrants who recently arrived in Germany [1]. Other countries in Europe have reported similar observations, including Austria, Belgium, France, Norway, Switzerland, and the United Kingdom [2]. Here we describe the first epidemiological data for 44 cases officially reported in Germany between 1 January and 30 September 2022, present phylogenetic analyses of 42 isolates respectively obtained from distinct cases and share suggestions for outbreak investigations and response that might be considered in other countries affected by this event.

## Descriptive analysis of the outbreak 

In Germany, diphtheria is generally laboratory-confirmed when the responsible pathogen such as* C. diphtheriae *or* C. ulcerans* is isolated in culture and either the *tox* gene coding for diphtheria toxin (DT) is detected by nucleic acid amplification (e.g. PCR) or evidence of production of DT is found by Elek–Ouchterlony immunoprecipitation (Elek) test (*tox*+ or toxigenic *C.* spp., respectively) [3]. This means that cases with proof of *tox*+* C.* spp. are also considered as confirmed cases despite a negative, pending, or not performed Elek test.

### Outbreak case definition

An outbreak investigation was initiated in August 2022 using the following working case definition. A confirmed case is any person who is either: (i) a migrant who has arrived in Germany and received laboratory confirmation of *tox*+* C. diphtheriae* on or after 1 January 2022, regardless of the manifestation, i.e. presenting with respiratory or cutaneous diphtheria or being asymptomatic or with unknown clinical data, or (ii) a close contact (as considered by the local health authority according to national recommendations [4]) of a confirmed case with laboratory confirmation of *tox*+* C. diphtheriae*, regardless of the country of origin and clinical presentation.

### Reported outbreak cases and their characteristics

**Figure 1:**
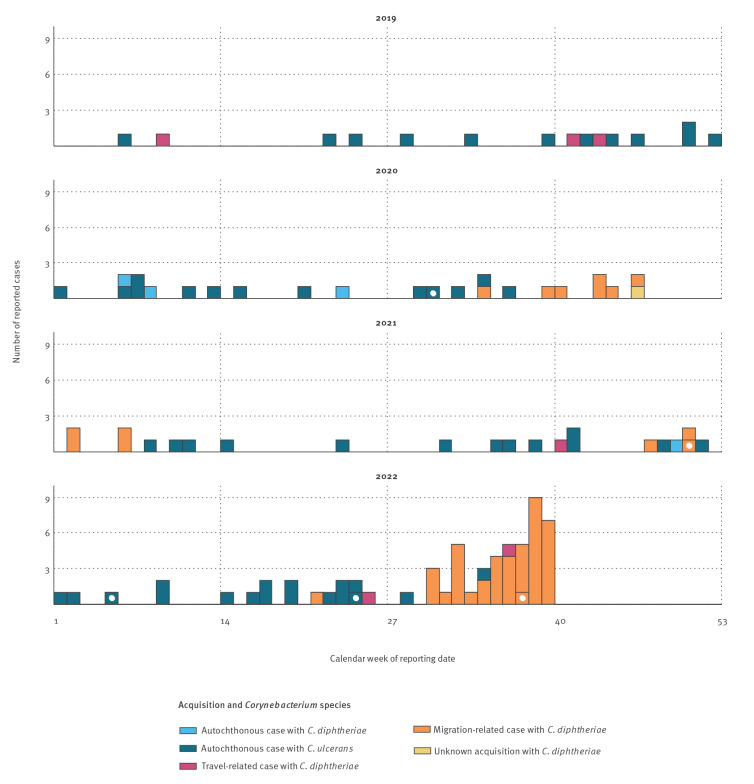
Weekly distributions of diphtheria cases fulfilling the German reference case definition^a^ reported to the national public health institute, Germany, 2019–2022^b^ (n = 123)

Between 1 January and 30 September 2022, the Robert Koch Institute (RKI), Germany’s national public health institute, received reports of 44 diphtheria cases who met the working outbreak case definition of a confirmed case (data up to 22 October 2022 to allow for delayed reporting and laboratory follow-up). Nineteen local health districts in seven of 16 federal states were affected. One case was reported in May 2022 (calendar week (CW) 21 2022), the other 43 starting from late July 2022 (CW 30–39 2022) (Figure 1). Of the 44 cases, 42 presented with cutaneous diphtheria, one with respiratory diphtheria and one was asymptomatic yet laboratory-confirmed. 

*C.*: *Corynebacterium*.

^a^ Case definitions in Germany are described in [3].

^b ^Data up to 22 October 2022.

Cases reported in 2022 only relate to the time period of 1 January to 30 September 2022. Cases marked with a white dot represent respiratory diphtheria (n = 5).

All outbreak-related cases were male with a median age of 18 years (interquartile range (IQR): 16–22) (Table 1). Date of symptom onset was available for 13 cases. For these cases, the respective dates of symptom onset ranged from 26 June to 23 September 2022. Although the date of symptom onset was only known in the minority of cases, anecdotal evidence indicated that in the case of wound infections these often had occurred weeks to months before arrival at reception centres in Germany (Christiane Wagner-Wiening, personal communication, October 2022). Several cutaneous lesions were co-infected with meticillin-resistant *Staphylococcus aureus* (MRSA) or *Streptococcus pyogenes*. The respiratory case was treated with diphtheria antitoxin (DAT).

**Table 1:** Demographic, clinical, and microbiological characteristics of outbreak-related diphtheria cases reported to the national public health institute, Germany, 1 January–30 September 2022^a^ (n = 44)

Case classification according to working outbreak case definition	Number of cases
Confirmed case	44
**Manifestation and pathogen**	
Respiratory diphtheria with *C. diphtheriae*	1
Cutaneous diphtheria with *C. diphtheriae*	42
No clinical confirmation or asymptomatic with *C. diphtheriae*	1
**Age group (years)**	
0–4	0
5–14	2
15–24	34
25–44	8
≥ 45	0
**Gender**	
Female	0
Male	44
Other/unknown	0
**Country of origin**	
Afghanistan	17
Syria	21
Tunisia	1
Yemen	1
Unknown	4
**Laboratory confirmation by National Consiliary Laboratory on Diphtheria**	
PCR+ and Elek+	44

*C. diphtheriae*: *Corynebacterium diphtheriae.*

^a ^Data up to 22 October 2022.

Most cases were detected by medical examinations in reception centres as it is mandatory for people living in a shared accommodation for migrants and asylum seekers to accept a clinical examination on communicable diseases. Vaccination status generally could not be established due to missing vaccination cards. Among the 44 cases, no human-to-human transmission within Germany could be confirmed. The asymptomatic case had been diagnosed during contact person management of the respiratory case and isolates from the two cases belonged to the same phylogenetic cluster (as shown further in the laboratory analysis and phylogeny section; isolates KL2203 and KL2189 respectively); nevertheless, it could not be determined whether this asymptomatic case was a secondary case infected by the respiratory case, whether they had the same source of infection or whether they got infected independently.

### Diphtheria cases 2019–2022

For comparison to the current situation in Germany with regard to diphtheria, Figure 1 depicts the diphtheria cases meeting the German reference case definition (see Supplement S1: Routine and intensified diphtheria surveillance in Germany) [3]. Between 2019 and 2021, cases of diphtheria in Germany were evenly distributed over the years, and average annual numbers of reported diphtheria cases were comparable to those reported in the yet incomplete year of 2022 for the local population and travellers (13.8 vs 18 for autochthonous transmission; 2.6 vs 2 for travel-related transmission), but not for migrants (2.8 vs 43 for migration-related transmission, excluding the asymptomatic case). Notably, all travel- and migration-related, i.e. imported, cases were caused by *C. diphtheriae,* while the majority of autochthonous cases were caused by *C. ulcerans*. In 2019, the first human-to-human transmission of cutaneous diphtheria caused by a toxigenic *C. diphtheriae* strain in almost 40 years within Germany was detected among siblings [5].

## Laboratory analysis and phylogeny

The German National Consiliary Laboratory on Diphtheria (NCLD) conducted *C. diphtheriae* strain identification, antimicrobial susceptibility testing, verified toxigenicity by real-time-PCR and a modified Elek test, and performed whole genome sequencing (WGS, see Supplement S2: Laboratory methods).

**Figure 2:**
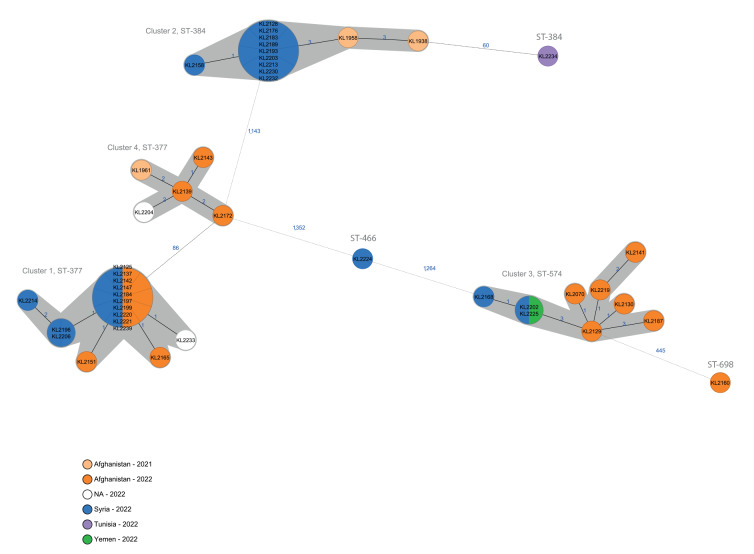
Minimum spanning tree of the cgMLST allelic profiles of *C. diphtheriae* isolates detected among migrants, Germany, 2021–2022^a^ (n = 45)

WGS data were analysed by multilocus sequence typing (MLST), using seven target loci, and also by core genome MLST (cgMLST), based on allele typing of 1,553 target loci. Figure 2 shows a minimum spanning tree (MST) based on cgMLST, including the genetic relationships as single-linkage (SL) allelic distances (AD), which display the shortest pairwise AD, rather than every absolute bilateral AD. The cgMLST analysis revealed six sequence types (ST) appearing on the phylogenetic tree as three singletons, and four genetically closely related distinct clusters, hereinafter numbered according to the number of isolates they comprised.

*C. diphtheriae*: *Corynebacterium diphtheriae*; cgMLST: core genome multi locus sequence typing; NA: not available (no information on country of origin); SL-AD: single-linkage allelic distance.

^a^ An ad hoc *C. diphtheriae* cgMLST scheme of 1,553 target loci was used for the cgMLST analysis.

Samples are colour-coded according to the case’s country of origin and collection year if information was available. The blue font is used for SL-ADs. Clusters with a SL-AD of ≤ 5 are shaded in grey.

Cluster 1 consisted of 16 isolates with ST-377 and was characterised by SL-ADs of 0–2 (maximum bilateral AD = 4). Cluster 2 comprised 12 isolates with ST-384, including two isolates from 2021, and had SL-ADs of 0–3 (maximum bilateral AD = 5). Cluster 3 comprised nine isolates with ST-574 and had SL-ADs of 0–3 (maximum bilateral AD = 7). Cluster 4 consisted of five isolates of ST-377 including one isolate from 2021 and had SL-ADs of 1–2 (maximum bilateral AD = 3); this cluster was separated by ≥ 86 SL-AD from Cluster 1, which was characterised by the same ST. The three singletons of ST-698, ST-466 and ST-384 showed an SL-AD of 445, 1,264 and 60 to the nearest cluster, respectively.

Clusters 1, 2, and 3 coincided with representative genome sequences of isolates from Austria and Norway, which were shared in the course of the investigation and in the European Centre for Disease Prevention and Control (ECDC) Rapid Risk Assessment [2] (data not shown). All isolates in Cluster 4 were phenotypically resistant to erythromycin and clindamycin. In line with the phenotypic results and recently published data from a similar cluster with ST-377 among migrants in Switzerland [6], our search for acquired resistance genes in Cluster 4 isolates found, among others, genes for resistance against macrolides/lincosamides and beta-lactams (*ermX*, *OXA-2*) (data not shown).

Comparison with sequences from diphtheria cases of migrants who had arrived in Germany in previous years revealed that two isolates matched with Cluster 2 and one isolate with Cluster 4 (pale orange colour in Figure 2).

## Analysis of migration routes

Countries of origin when known, were mostly Syria (n = 21) and Afghanistan (n = 17), however, there were also cases from Tunisia and Yemen (i.e. one from each country). Information on transit countries on route to Germany was available for 23 of the 44 cases as well as for the three cases from 2021 who were infected by strains belonging to ST clusters identified in the current outbreak (Table 2). Of the resulting 26 individuals, 22 mentioned having transited through one or more countries along the Balkan route, i.e. Albania, Bosnia and Herzegovina, Bulgaria, Croatia, North Macedonia, Romania, Serbia, and Slovenia. Two persons mentioned transiting through Belarus and Poland, i.e. the Belarus route; one of them initially also transited along the Balkan route. Cases mentioning transit countries along the Balkan route, including the three cases from 2021, are mainly distributed among Clusters 1, 2, and 4. In contrast, Cluster 3 contains only one case who transited through Balkan countries. Although another case in this cluster travelled via the Belarus route, it should be noted that most cases (7 of 9) in Cluster 3 had little details available about their journey to Germany. Investigations by local health authorities revealed that migrants were *en route* often for months, living under very poor hygienic conditions. The majority of them stayed in migration camps or in forests in the Balkans. Injuries acquired while fleeing were not treated due to lack of access to healthcare services.

**Table 2:** Migration routes, including transit countries, named by diphtheria cases among migrants in Germany, 2021–2022^a^ (n = 47)

Identifier at NCLD	Year of reporting	Country of origin	ST	Cluster	Migration route to Germany	Balkan route
KL2125	2022	Afghanistan	377	1	Afghanistan	Unknown
KL2142	2022	Afghanistan	377	1	Afghanistan	Unknown
KL2147	2022	Afghanistan	377	1	Afghanistan	Unknown
KL2196	2022	Syria	377	1	Syria	Unknown
KL2220	2022	Syria	377	1	Syria	Unknown
KL2137	2022	Afghanistan	377	1	Afghanistan, via Pakistan, Iran, Türkiye, Greece, Serbia, Hungary, Austria	Yes
KL2151	2022	Afghanistan	377	1	Afghanistan, via Bulgaria	Yes
KL2165	2022	Afghanistan	377	1	Afghanistan, via Bosnia and Herzegovina, Serbia	Yes
KL2197	2022	Syria	377	1	Syria, via Serbia	Yes
KL2199	2022	Syria	377	1	Syria, via Iraq, Türkiye, Greece, Albania, Serbia	Yes
KL2206	2022	Syria	377	1	Syria, via Bosnia and Herzegovina, Serbia	Yes
KL2184	2022	Syria	377	1	Syria, via Türkiye, North Macedonia, Serbia, Czechia	Yes
KL2214	2022	Syria	377	1	Syria, via Türkiye, Bulgaria, North Macedonia, Serbia, Slovenia	Yes
KL2233	2022	Unknown	377	1	via Syria, Türkiye, Greece, North Macedonia, Serbia, Hungary, Czechia	Yes
KL2221	2022	Afghanistan	377	1	Afghanistan	Unknown
KL2239	2022	Syria	377	1	Syria, via Türkiye, Bulgaria, Serbia, Hungary, Austria	Yes
KL1938	2021	Afghanistan	384	2	Afghanistan, via Iran, Türkiye, Greece, Romania, Serbia, Belarus, Poland	Yes
KL1958	2021	Afghanistan	384	2	Afghanistan, via Syria, Türkiye, Greece	Yes
KL2128	2022	Syria	384	2	Syria	Unknown
KL2158	2022	Syria	384	2	Syria	Unknown
KL2176	2022	Syria	384	2	Syria	Unknown
KL2189	2022	Syria	384	2	Syria	Unknown
KL2203	2022	Syria	384	2	Unknown	Unknown
KL2183	2022	Syria	384	2	Syria, via Türkiye, Bulgaria, Serbia, Hungary, Slovakia, Czechia	Yes
KL2193	2022	Syria	384	2	Syria, via Türkiye, Serbia, Croatia	Yes
KL2213	2022	Syria	384	2	Syria, via Türkiye, Bulgaria, North Macedonia, Serbia, Slovenia, Czechia	Yes
KL2230	2022	Syria	384	2	Unknown	Unknown
KL2232	2022	Syria	384	2	Syria, via Türkiye, Bulgaria, Serbia, Hungary, Austria	Yes
KL2070	2022	Afghanistan	574	3	Afghanistan	Unknown
KL2129	2022	Afghanistan	574	3	Afghanistan	Unknown
KL2130	2022	Afghanistan	574	3	Afghanistan	Unknown
KL2141	2022	Afghanistan	574	3	Afghanistan, via Bulgaria	Yes
KL2168	2022	Syria	574	3	Syria	Unknown
KL2187	2022	Afghanistan	574	3	Afghanistan, via Türkiye, Belarus, Poland	No
KL2202	2022	Yemen	574	3	Yemen, via Syria	Unknown
KL2219	2022	Afghanistan	574	3	Afghanistan, via Türkiye	Unknown
KL2225	2022	Syria	574	3	Unknown	Unknown
KL1961	2021	Afghanistan	377	4	Afghanistan, via Pakistan, Iran, Türkiye, Bulgaria, Serbia, Hungary, Austria	Yes
KL2139	2022	Afghanistan	377	4	Afghanistan, via Bulgaria	Yes
KL2143	2022	Afghanistan	377	4	Afghanistan	Unknown
KL2172	2022	Afghanistan	377	4	Afghanistan, via Bulgaria	Yes
KL2204	2022	Unknown	377	4	Unknown	Unknown
KL2160	2022	Afghanistan	698	None	Afghanistan, via Serbia, Hungary, Austria	Yes
KL2224	2022	Syria	466	None	Syria, via Balkan route	Yes
KL2234	2022	Tunisia	384	None	Tunisia, via Italy, Austria	No
KL2249	2022	Unknown	Pending	Pending	Unknown	Unknown
KL2257	2022	Unknown	Pending	Pending	Unknown	Unknown

NCLD: German National Consiliary Laboratory on Diphtheria; ST: sequence type.

^a^ Data up to 22 October 2022.

This table comprises all cases of diphtheria reported to Germany’s national public health institute, from 1 January to 30 September 2022, that fulfil the working case definition of this outbreak investigation (n = 44) as well as additional cases from 2021 whose isolates belong to the same STs or clusters relevant in this outbreak investigation (n = 3). The migration route starts with the country of departure and continues via transit countries if such countries were reported.

## Discussion

Diphtheria is a vaccine-preventable disease caused by DT producing, i.e. toxigenic, *C. *spp. Diphtheria predominantly presents as a respiratory or a cutaneous manifestation. In Germany, the majority of cases notified in recent years have been cutaneous diphtheria caused by zoonotic, toxigenic *C. ulcerans* [7]. This is likely due to incidental findings following improvements in the microbiological diagnostic methods [8] and its wider use. However, since July 2022, we observed an unprecedented increase of diphtheria cases caused by toxigenic *C. diphtheriae* among migrants who recently arrived in Germany.

Upon investigating this event, we considered among others the following four aspects. Firstly, a change in the incidence of diphtheria in the countries of origin. For 2021, Afghanistan reported 61 cases to the World Health Organization (WHO), after several years with zero or single-digit case counts, or no reporting [9]. Whether the increase in 2021 is due to an increased circulation of *C.* spp. remains unknown. We also lack information on the epidemiology of cutaneous diphtheria in Afghanistan and Syria in 2022. Coverage of a diphtheria-containing vaccine represented by the third dose of diphtheria, tetanus and pertussis vaccines (DTP3) was 66% in Afghanistan, 78% in Iraq, and 48% in Syria, as WHO and United Nations Children's fund (UNICEF) estimated for 2021 [10].

Secondly, an increase in the number of migrants arriving in Germany. In 2022, more migrants came to Germany than in 2021, but not in the order of magnitude as imported diphtheria cases increased [11-13]. A proxy for the number of arriving migrants is the number of first asylum applications. In the third quarter (July–September) of 2022, approximately 4% more Afghans and 20% more Syrians applied for asylum in Germany for the first time, compared with the third quarter of 2021. Interestingly, no diphtheria case in this outbreak has been reported among migrants from Iraq as country of origin although Iraqis are another main group seeking asylum in Germany [13]. Migrants from Iraq to Europe more commonly arrive via the Belarus route which passes via Moldova, Ukraine, Belarus, and Poland [14].

Thirdly, a possible detection bias due to increased diagnostics of skin lesions in the wake of the monkeypox (MPX) outbreak [15]. This outbreak and increased awareness to detect and diagnose skin lesions especially among young males since May 2022 might have helped to detect the diphtheria upsurge early. Local health authorities reported that several diphtheria cases were diagnosed after initial suspicion of MPX which was ruled out.

Fourthly we considered, human-to-human transmission in reception centres in Germany. Germany’s national public health institute continuously raises awareness and advises local and federal health authorities to follow the general recommendations for the management of diphtheria cases and contact persons as well as the particular recommendation for outbreaks of infectious diseases in shared accommodations [4,16]. Among the 44 cases reported by 19 different local health authorities until 30 September 2022, no secondary case could be confirmed. Nevertheless, in October 2022, after the data cut-off time for this report, two local health authorities reported one secondary case each deriving from a respiratory diphtheria case.

All the listed aspects might have contributed to the remarkable upsurge of migration-related diphtheria cases but do not fully explain it. Our sequencing and epidemiological data as well as information regarding the migration routes confirm that the majority of cases most likely acquired *C. diphtheriae* during migration, particularly along the Balkan route. Nevertheless, individual persons could also have already been colonised with *C. diphtheriae* in their country of origin, been injured *en route*, and only then developed cutaneous diphtheria.

That cases are imported rather than occurring as secondary cases within Germany is supported by further evidence. For one, in each cluster, individuals came from different countries of origin and then resided in different cities in Germany. Also, isolates from Austria matched with Clusters 1, 2, and 3, and an isolate from Norway matched with Cluster 1. Additionally, the molecular clock also indicates an origin abroad; in a recent publication of a similar outbreak situation in Switzerland, a molecular clock model of 1.67 × 10 ^− 6^ substitutions per site and year [17] was used in a phylogeny based on single nucleotide polymorphism (SNP) to date back the occurrence of 1 SNP between two isolates to ≥ 6 weeks, 2 SNPs to 5 months and 5 SNPs to 1 year [6]. In our study, the phylogenetic relationship was analysed by cgMLST, based on AD, not SNPs, which have been shown to be within a similar range as the used cgMLST scheme [18], although they generally tend to be slightly smaller in numbers than corresponding SNP distances. Therefore, the model proposed in the Swiss study [6] can be used as an approximation. Maximum AD in Clusters 1, 2, 3, and 4 were 4, 5, 7, and 3, respectively, suggesting transmission events within the last year.

Potential limitations of this study are, besides those mentioned earlier, that migration route is not known for all cases, and that time between arrival in reception centres and medical examination is not reported and therefore human-to-human transmission in the accommodation could remain unidentified.

## Conclusions 

In 2022 three times more diphtheria cases were reported in a 10-week period (CW 30 to 39) than in the 3 previous years altogether. This event, as per the ECDC definition [19], constitutes an outbreak. Based on our data, it seems likely that this outbreak originated abroad, not in Germany, and that there are multiple sources of infection, mainly along the Balkan route. Nevertheless, no epicentres have been identified yet. Considering that 19 of 23 cases from 2022 with known migration route came along the Balkan route and since similar cases occurred in other European countries, this outbreak likely involves multiple countries. 

Based on our findings, it seems crucial to identify the source(s) of this outbreak. Outbreak investigations in further countries could provide necessary data on the epidemiology, phylogeny and detailed migration routes. Analyses from pooled molecular data from different countries may also help narrow down where infections might have been acquired. In this respect, a study on diphtheria outbreaks in a Swiss asylum centre has just been published [6], with identification of a number of identical STs to our study. Moreover, active case finding is necessary, not only in Germany and other destination countries, but also in the main countries of origin and countries along the Balkan route. In contrast to severe respiratory diphtheria, cutaneous diphtheria may have unspecific symptoms and be masked by co-infections but is a potential source for secondary cases. Case and contact management should be in accordance with respective national guidelines. Laboratory preparedness should also be increased in possibly affected countries, including personnel and reagents. Laboratory confirmation by PCR and Elek test is important to reveal the true magnitude of the ongoing outbreak, and sequencing of isolates from both migration-related cases as well as autochthonous cases is necessary for further in-depth analysis. Altogether, this could contribute to effectively respond to this multi-country diphtheria outbreak and to implement infection prevention and control measures such as targeted and efficient vaccination campaigns.

## Supplementary Data

81000 Supplementary Material
