# Supplementary material for: Outbreak of imported diphtheria with Corynebacterium diphtheriae among migrants arriving in Germany, 2022
Source: Euro Surveill. 2022 Nov 17;27(46):2200849. doi: 10.2807/1560-7917.ES.2022.27.46.2200849 (PMC9673234; doi:10.2807/1560-7917.ES.2022.27.46.2200849)
Supplement: Supplementary Material [file 2200849_SupplementaryMaterial.pdf]

Supplementary Material

This supplementary material is hosted by *Eurosurveillance* as supporting information alongside the article **"Outbreak of imported diphtheria with *Corynebacterium diphtheriae* among migrants arriving in Germany, 2022"** on behalf of the authors who remain responsible for the accuracy and appropriateness of the content. The same standards for ethics, copyright, attributions and permissions as for the article apply. Supplements are not edited by Eurosurveillance and the journal is not responsible for the maintenance of any links or email addresses provided therein.

S1: Routine and intensified diphtheria surveillance in Germany

In Germany, clinical suspicion, proof of diphtheria disease or death as well as the identification of DT producing *Corynebacterium (C.)* spp. are notifiable to the local health authority [1]. Case definition categories comprise [2]:

- clinical disease with epidemiological link,
- clinically and laboratory-confirmed disease,
- laboratory-confirmed infection without clinical confirmation, and
- laboratory-confirmed infection with unknown clinical status.

The reference definition for official statistics includes cases of the first two categories [2]. This currently used German case definition became effective in 2017 [3].

Most diphtheria-related strains isolated in Germany are analysed for confirmation and toxigenicity testing at the National Consiliary Laboratory on Diphtheria (NCLD).

Routine diphtheria surveillance in Germany is based on a standardised data entry mask for diphtheria cases that is mandatory for any case that is notifiable and to be reported by law.

An intensified diphtheria surveillance has been established in Germany in 2019 and shall provide additional information, e.g. on diphtheria antitoxin (DAT) use, antimicrobial resistances, contact persons and travel history, if applicable. Participation in the intensified diphtheria surveillance is voluntary and comprises a case-based questionnaire.

S2: Laboratory methods

After the initial swab culture on Hoyle's Tellurite agar (BD Life Sciences, Heidelberg, Germany) and Columbia blood agar (Oxoid, Wesel, Germany) for 24-48 hours at 37°C and in 5% CO_2, *C. diphtheriae* strains were identified by biochemical differentiation (API CORYNE; bioMerieux SA, Marcy l´Etoile, France) and matrix-assisted laser desorption/ionisation time-of-flight mass spectrometry (MALDI-TOF MS; MALDI Biotyper; Bruker Daltonics, Bremen, Germany) [4].

Antimicrobial susceptibility testing was performed on Mueller-Hinton-Agar (supplemented with 5% horse blood) by Etest (bioMerieux SA, Marcy l´Etoile, France) after overnight incubation at 37°C and in 5% CO_2. Minimum inhibitory concentrations were determined according to the guideline of the European Committee on Antimicrobial Susceptibility Testing (EUCAST), version 12 [5], and the Clinical and Laboratory Standards Institute (CLSI) M45 2015 guidelines [6].

Toxigenicity was verified by real-time-PCR [7] and a modified Elek test [8].

Whole genome sequencing (WGS) was performed after DNA extraction from isolates on blood agar plates [9] and Illumina DNA preparation on a NextSeq (Illumina, San Diego, CA, USA) with 2 x 75 bp. For a phylogenetic analysis, multi locus sequence typing (MLST) and core genome (cg) MLST were performed in Ridom SeqSphere+ (Ridom, Munster, Germany) using the 7 MLST target regions *atpA*, *dnaE*, *dnaK*, *fusA*, *leuA*, *odhA* described in [10] and an ad-hoc *C. diphtheriae* cgMLST scheme of 1553 target loci described in [9] in comparison to genome sequences of isolates sequenced earlier. Duplicate isolates from the same persons were excluded after it was verified that they were genetically identical. WGS assemblies were searched for acquired resistance genes using the card database [11], using abricate [12] (data not shown).

S3 – Supplementary Table: Accession numbers of WGS data at NCBI under bioproject PRJNA898270

Sample ID	Biosample accession	SRA accession
KL1938	SAMN31602458	SRR22189726
KL1958	SAMN31602459	SRR22189725
KL1961	SAMN31602460	SRR22189714
KL2070	SAMN31602461	SRR22189703
KL2125	SAMN31602462	SRR22189692
KL2128	SAMN31602463	SRR22189686
KL2129	SAMN31602464	SRR22189685
KL2130	SAMN31602465	SRR22189684
KL2137	SAMN31602466	SRR22189683
KL2139	SAMN31602467	SRR22189682
KL2141	SAMN31602468	SRR22189724
KL2142	SAMN31602469	SRR22189723
KL2143	SAMN31602470	SRR22189722
KL2147	SAMN31602471	SRR22189721
KL2151	SAMN31602472	SRR22189720
KL2158	SAMN31602473	SRR22189719
KL2160	SAMN31602474	SRR22189718
KL2165	SAMN31602475	SRR22189717
KL2168	SAMN31602476	SRR22189716
KL2172	SAMN31602477	SRR22189715
KL2176	SAMN31602478	SRR22189713
KL2183	SAMN31602479	SRR22189712
KL2184	SAMN31602480	SRR22189711
KL2187	SAMN31602481	SRR22189710
KL2189	SAMN31602482	SRR22189709
KL2193	SAMN31602483	SRR22189708
KL2196	SAMN31602484	SRR22189707
KL2197	SAMN31602485	SRR22189706
KL2199	SAMN31602486	SRR22189705
KL2202	SAMN31602487	SRR22189704
KL2203	SAMN31602488	SRR22189702
KL2204	SAMN31602489	SRR22189701
KL2206	SAMN31602490	SRR22189700
KL2213	SAMN31602491	SRR22189699
KL2214	SAMN31602492	SRR22189698
KL2219	SAMN31602493	SRR22189697
KL2220	SAMN31602494	SRR22189696
KL2221	SAMN31602495	SRR22189695
KL2224	SAMN31602496	SRR22189694
KL2225	SAMN31602497	SRR22189693

Sample ID	Biosample accession	SRA accession
KL2230	SAMN31602498	SRR22189691
KL2232	SAMN31602499	SRR22189690
KL2233	SAMN31602500	SRR22189689
KL2234	SAMN31602501	SRR22189688
KL2239	SAMN31602502	SRR22189687

References

1. Gesetz zur Verhütung und Bekämpfung von Infektionskrankheiten beim Menschen (Infektionsschutzgesetz - IfSG) [Infection Protection Law] [Internet]. 2020. Available from: https://www.gesetze-im-internet.de/ifsg/BJNR104510000.html
2. Robert Koch Institute (RKI). Falldefinitionen des Robert Koch-Instituts zur Übermittlung von Erkrankungs- oder Todesfällen und Nachweisen von Krankheitserregern [Case definitions of RKI for reporting of cases of disease or death and confirmation of pathogens]. 2019; Available from: www.rki.de/falldefinitionen
3. Robert Koch Institute (RKI). Änderungen des Infektionsschutzgesetzes, Juli 2017 [Change of Infection Protection Law, July 2017]. Epidemiol Bull [Internet]. 2017;(31):297–314. Available from: https://www.rki.de/DE/Content/Infekt/EpidBull/Archiv/2017/Ausgaben/31_17.pdf
4. Konrad R, Berger A, Huber I, Boschert V, Hörmansdorfer S, Busch U, et al. Matrix-assisted laser desorption/ionisation time-offlight (MALDI-TOF) mass spectrometry as a tool for rapid diagnosis of potentially toxigenic Corynebacterium species in the laboratory management of diphtheriaassociated bacteria. Eurosurveillance [Internet]. 2010;15(43):1–5. Available from: http://dx.doi.org/10.2807/ese.15.43.19699-en
5. The European Committee on Antimicrobial Susceptibility Testing (EUCAST). Breakpoint tables for bacteria, version 12.0 [Internet]. 2022 [cited 2022 Oct 24]. Available from: https://www.eucast.org/clinical_breakpoints
6. Clinical and Laboratory Standards Institute (CLSI). M45 Methods for Antimicrobial Dilution and Disk Susceptibility Testing of Infrequently Isolated or Fastidious Bacteria. 2015.
7. Schuhegger R, Lindermayer M, Kugler R, Heesemann J, Busch U, Sing A. Detection of toxigenic Corynebacterium diphtheriae and Corynebacterium ulcerans strains by a novel real-time PCR. J Clin Microbiol. 2008;46(8):2822–3.
8. Melnikov VG, Berger A, Sing A. Detection of diphtheria toxin production by toxigenic corynebacteria using an optimized Elek test. Infection [Internet]. 2022;(0123456789). Available from: https://doi.org/10.1007/s15010-022-01903-x
9. Dangel A, Berger A, Konrad R, Bischoff H, Sing A. Geographically diverse clusters of nontoxigenic corynebacterium diphtheriae infection, Germany, 2016–2017. Emerg Infect Dis. 2018;24(7):1239–45.
10. Bolt F, Cassiday P, Tondella ML, DeZoysa A, Efstratiou A, Sing A, et al. Multilocus sequence typing identifies evidence for recombination and two distinct lineages of Corynebacterium diphtheriae. J Clin Microbiol. 2010;48(11):4177–85.
11. McArthur AG, Waglechner N, Nizam F, Yan A, Azad MA, Baylay AJ, et al. The comprehensive antibiotic resistance database. Antimicrob Agents Chemother. 2013;57(7):3348–57.
12. Seemann T. ABRicate. Mass screening of contigs for antimicrobial resistance or virulence genes. [Internet]. 2020 [cited 2022 Oct 22]. Available from: https://github.com/tseemann/abricate
